# Dural arteriovenous fistula with progressive dementia and parkinsonism: Two case reports and a literature review

**DOI:** 10.1097/MD.0000000000035782

**Published:** 2023-11-10

**Authors:** Jiangbo Xie, Tingting Zhang, Ying Zhang, Weiwei Wu, Peichun Li, Xuezheng Zhang

**Affiliations:** a Department of Neurology, Weifang Traditional Chinese Hospital, Weifang, China.

**Keywords:** digital subtraction angiography, dural arteriovenous fistula, Parkinsonism, progressive dementia

## Abstract

**Rationale:**

Dural arteriovenous fistulas (DAVFs) are rare cerebral abnormal arteriovenous anastomoses. *It* is *uncommon for* DAVFs with parkinsonism and *dementia, so it* is easily misdiagnosed. Neuroimaging examinations show that multifocal DAVFs are related to venous thrombosis and white matter changes, suggesting that cerebral circulatory disorders caused by venous hypertensive encephalopathy lead to dementia in patients. Digital subtraction angiography confirmed the diagnosis and subsequent treatment of DAVFs.

**Patient concerns:**

We report 2 cases, one caused by bilateral white matter lesions and the other caused by bilateral thalamus lesions. Their symptoms are all manifested as progressive dementia and parkinsonism.

**Diagnosis:**

They were diagnosed with dural arteriovenous fistulas by digital subtraction angiography.

**Outcomes:**

The first patient developed progressive cognitive impairment, 6 months later, the patient developed bedridden, incontinence, and severe cognitive function.The second patient became increasingly bedridden 3 months after discharge and died of aspiration pneumonia.

**Lessons:**

There are few reports of progressive dementia and parkinsonism in DAVF patients, and neurologists should be vigilant to avoid misdiagnosing DAVF.

## 1. Introduction

Dural arteriovenous fistulas (DAVFs) are part of a category of intracranial vascular malformations, accounting for approximately 10% to 15% of all intracranial vascular malformations.^[1]^ Although the clinical manifestations of intracranial DAVFs are diverse, typical symptoms include pulsating tinnitus, eye paralysis, exophthalmos, headache, cognitive impairment, and intracranial hemorrhage or infarction.^[2]^ However, there are few reports of progressive dementia with parkinsonism. Here, we report 2 patients with DAVFs with concurrent progressive dementia with parkinsonism.

## 2. Case report

### 2.1. Case1

A 70-year-old right-handed woman experienced gradual slow responsiveness and sluggishness for 6 months. The initial examination in a local hospital found lacunar infarction 4 months prior to examination. Three months before admission, the patient symptoms gradually worsened, and tremors appeared. Specifically, involuntary tremor of the right upper and hind limbs, accompanied by slow walking, unstable gait, and frequent falls, occurred. Worryingly, muscle tension of the limbs gradually increased, and the patient cognitive skills began to lapse. One week before admission, the patient went to a local clinic, where she was prescribed Madopar, but the symptoms did not improve. She gradually lost the ability to walk and was then admitted to our hospital for treatment. The patient had a history of hypertension for 6 years and had no history of head trauma, skull surgery, or central nervous system infection.

Neurological examination showed a clear mind, but the patient had reduced communication skills; this reduction in communication skills was accompanied by severely decreased short-term memory and reduced basic calculation ability. The patient had an elementary school education with a mini-mental state examination score of 18/30. The examination also showed resting tremor of the upper limbs, increased muscle tone of the limbs, and positive bilateral pathological signs.

**Figure 1.**
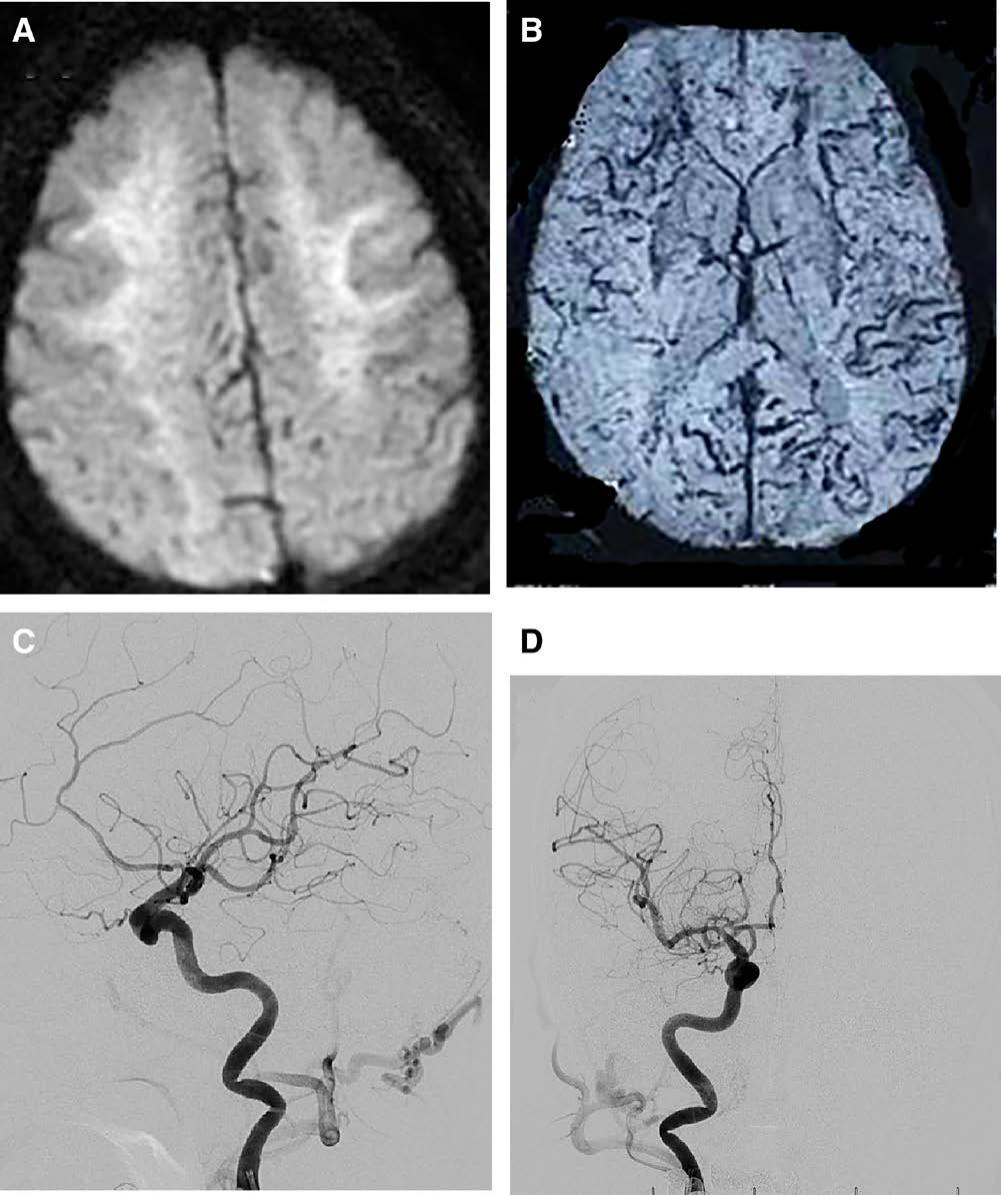
(A) Brain magnetic resonance imaging (MRI) indicated extensive white matter lesions in both cerebral hemispheres. (B) Craniocerebral susceptibility weighted imaging (SWI) indicated alterations in venous dilation. (C) Digital subtraction angiography (DSA) indicated that the upper and lower sagittal sinuses and bilateral transverse sinuses were affected. (D) DSA indicated that the upper and lower sagittal sinuses and bilateral transverse sinuses were affected.

The patient showed progressive cognitive decline, accompanied by slow movements, bilateral upper extremity tremors, and typical Parkinsonian symptoms, with increased muscle tone in the extremities. After admission, there were no obvious abnormalities in waist magnetic resonance imaging (MRI) findings, dynamic electrocardiogram results, thyroid function test results, red blood cell sedimentation rate, folic acid levels, vitamin B12 levels, syphilis presence, or acquired immune deficiency syndrome test results. Lumbar puncture indicated a normal cerebrospinal fluid pressure (190 mm H_2_O), and the results of a routine examination of cerebrospinal fluid cells and biochemical attributes were normal; however, an electroencephalogram showed slow wave activity, and brain MRI indicated extensive white matter lesions in both cerebral hemispheres (Fig. 1A and B). To determine a clear diagnosis, digital subtraction angiography (DSA) indicated that the upper and lower sagittal sinuses and bilateral transverse sinuses were affected (Fig. 1C and D). Considering DAVF, we recommended endovascular treatment. The patient family declined treatment due to financial concerns. One month after being discharged from the hospital, we followed up with the patient, and her cognitive impairment had worsened. Six months later, the patient developed bedridden, incontinence, and severe cognitive function.

### 2.2. Case 2

The patient was a 67-year-old male who began to experience cognitive decline 10 months prior to examination. The patient consistently repeated phrases, discussed a single problem repeatedly, and failed to return objects to original positions, all of which were accompanied by involuntary tremor of the upper limbs. At 6 months, the patient condition worsened. He reported pain and discomfort in the occiput and back of the neck, which was accompanied by reduced speech, increased lethargy, and inability to wash or eat actively; therefore, family supervision was required. The patient also showed diminished memory, whereby he would easily lose his way in previously familiar environments and would not be able to find his way home. In all other aspects, the patient was healthy.

**Figure 2.**
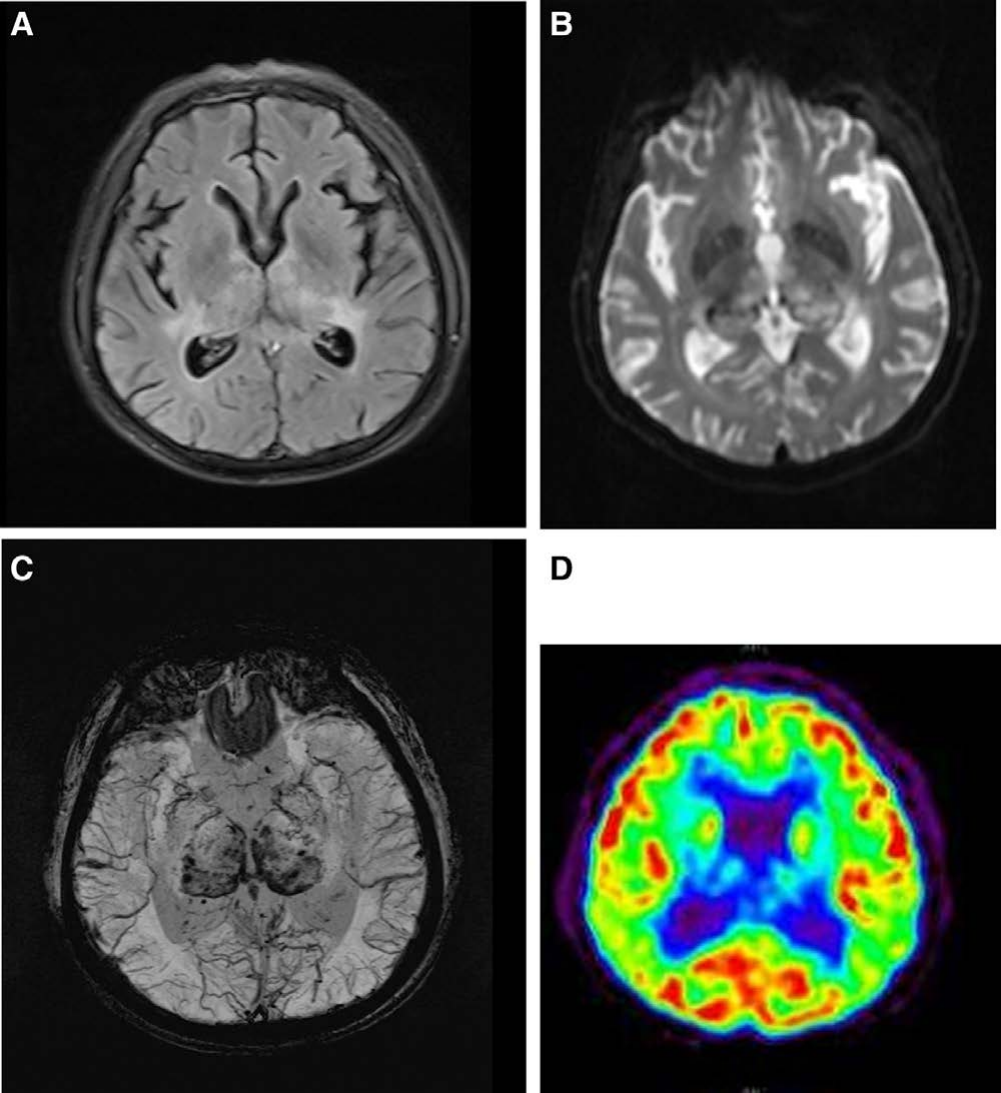
(A) Plain magnetic resonance imaging (MRI) scan result showed that an abnormal signal was symmetrical in the bilateral thalamus. (B) Diffusion weighted imaging (DWI) result showed that an abnormal signal was symmetrical in the bilateral thalamus. (C) Craniocerebral susceptibility weighted imaging (SWI) indicated bilateral thalamus microhemorrhage and alterations in venous dilation. (D) Positron emission tomography-computed tomography (PET-CT) examination revealed decreased symmetrical density of the bilateral thalamus and lowered glucose metabolism.

Neurological examination showed significantly reduced cognitive and calculation abilities—the patient was unable to answer questions and had an mini-mental state examination score of 10/30. The patient also presented with an obvious tremor of the upper limbs. Lumbar puncture indicated normal cerebral spinal fluid pressure (150 mm H_2_O), and routine examination of cerebrospinal fluid cells and biochemical attributes indicated no abnormalities; however, routine blood examination indicated that the patient was cytomegalovirus-IgG positive, as did the results of a high-throughput genetic test report. The likely diagnosis was considered to be cytomegalovirus encephalitis, and immediate antiviral treatment was given; however, the patient symptoms did not improve with this treatment. Plain MRI scan and diffusion weighted imaging results showed that an abnormal signal was symmetrical in the bilateral thalamus (Fig. 2A and B). Craniocerebral susceptibility weighted imaging (SWI) indicated bilateral thalamus microhemorrhage and alterations in venous dilation (Fig. 2C). On the 5th day of hospitalization, positron emission tomography-computed tomography examination revealed decreased symmetrical density of the bilateral thalamus and lowered glucose metabolism (Fig. 2D). The patient treatment regimen proved ineffective, and he was discharged to a superior hospital. DSA examination showed that the internal cerebral vein, vein of Galen and straight sinus were not clearly displayed. The diagnosis was corrected to DAVF combined with intracranial venous thrombosis. Unfortunately, the patient DSA image was lost, and after 3 months the patient gradually became bedridden and died of aspiration pneumonia.

## 3. Discussion

The most common onset age of DAVF is 60 to 70 years, while the ratio of affected males to females is 1:1.65.^[1]^ DAVFs primarily affect the external carotid artery but, to a lesser extent, can also affect the meningeal branches of the internal carotid and vertebral arteries.^[3]^ The etiology includes trauma, inflammation, and iatrogenic injury, among others; these issues result in increased venous network trafficking and eventual arteriovenous fistula formation. Fistula formation may be directly related to a decrease in blood vessel wall elasticity and an increase in fragility and circuit expansion, as well as alterations in blood flow caused by changes in estrogen levels. At the same time, congenital fibromuscular hyperplasia, vascular malformations, and other congenital factors cannot be ignored.

Patients with DAVFs have many clinical manifestations, which depend on the location and method of venous drainage.^[1,2,4]^ DAVFs often involve the transverse and sigmoid sinuses, followed by the cavernous and superior sagittal sinuses. Common symptoms include mild headache, orbital congestion, pulsatile tinnitus, and ophthalmoplegia, whereas more serious symptoms include neurological deficits and acute intracranial hemorrhage.^[1,2]^ Because of the wide and variable range of clinical symptoms and imaging features, DAVFs are easily misdiagnosed as other diseases.^[5]^

There are reports in the literature that dementia is the main symptom of DAVF.^[6]^ The pathogenesis of dementia in DAVF patients is still unclear. At present, there are 2 pathogenic mechanisms of cortical dementia and thalamic dementia. Cortical dementia is an obstruction caused by the absorption of cerebrospinal fluid, venous hypertension or direct backflow of DAVFs to the medullary vein, which leads to arterialization of the medullary vein, subsequently leading to venous congestion.^[6]^ As supported by our second case, it is currently believed that thalamic dementia is mainly secondary to thalamic venous congestion and ischemia due to impaired deep venous drainage. This flow usually returns from the aponeurotic vein to the straight sinuses and veins of Galen, then the intracerebral veins and basal veins. The thalamic veins from the upper and medial parts drain into the internal cerebral veins, while the thalamic veins from the lower and lateral parts drain into the basilar veins. These findings indicated impaired thalamus function.

The mechanism by which DAVFs cause parkinsonism is unclear. One explanation is that parkinsonism manifests due to impaired deep vein drainage and insufficient perfusion of the basal ganglia, consistent with our second case report. Another is that hypoperfusion of the frontal lobe caused by venous hypertension is considered to be causative of Parkinson disease in patients with DAVF.^[3]^ In this case, the extensive bilateral white matter lesions in our first case fit the latter explanation. Yamanouchi and Nagura^[7]^ studied patients with vascular Parkinson disease that were also thought to be caused by frontal white matter lesions and found that DAVFs could be easily confused with parkinsonism.

At present, brain and enhanced CT scans are not able provide data that can lead to a definitive diagnosis of DAVF but can show secondary manifestations, such as vasodilation, cerebral hemorrhage, hydrocephalus, cerebral edema, and calcification.^[8]^ Also, MRI is able to show abnormal images of blood vessels. When DAVFs are accompanied by cortical venous reflux, vasodilatation of the cortical vascular system, meningeal or medullary vessels, and sinuses occurs.^[9]^ SWI is able to detect hypoxic blood and depict vein structures in blood vessels; SWI findings can be used to identify venous vasodilation caused by prolonged intracranial blood flow, venous congestion, and functional obstruction caused by DAVFs.^[10]^

DSA can determine the location of the fistula, feeding artery, draining venous sinus, and cortical venous reflux; these features are which are the main bases for the diagnosis, classification, and treatment of DAVFs. Overall, DSA examination can allow a quick diagnosis, subsequently leading to prompt disease treatment.

Treatments for DAVF include endovascular embolization, vascular compression, surgery and stereotactic treatment. With the development of modern materials and devices, endovascular treatment has become the main method for DAVF treatment.

The limitation of this study is that neither of these 2 patients received further endovascular treatment and thus experienced deterioration.

## 4. Conclusion

In conclusion, DAVFs accompanied by dementia and parkinsonism are easily misdiagnosed. Therefore, timely diagnosis is very important to improve treatment efficacy. For progressive dementia with Parkinson disease, when brain MRI scans show white matter lesions, the possibility of DAVF should be considered. Complete imaging examinations are needed concurrent with DSA examination and endovascular treatment to improve patients’ overall prognosis.

## Acknowledgments

The authors appreciate the patients cooperation sincerely.

## Author contributions

**Conceptualization:** Tingting Zhang.

**Formal analysis:** Weiwei Wu.

**Methodology:** Ying Zhang.

**Project administration:** Peichun Li.

**Writing – original draft:** Jiangbo Xie.

**Writing – review & editing:** Xuezheng Zhang.
